# A case presentation of patient from northern China with endomyocardial fibrosis

**DOI:** 10.1186/s12872-019-01305-2

**Published:** 2019-12-26

**Authors:** Yonggang Yuan, Yingkai Li, Zesheng Xu

**Affiliations:** Department of Cardiology, Cangzhou Central Hospital, Cangzhou, 061000 Hebei China

**Keywords:** Endomyocardial fibrosis, Heart failure, Echocardiography, Calcification, Ventricular fibrosis

## Abstract

**Background:**

Endomyocardial fibrosis (EMF) is a rare condition and a major cause of death in tropical countries. The etiology of EMF remains elusive, and no specific treatment has been developed yet, therefore it carries poor prognosis.

**Case presentation:**

An 81-year-old male Chinese patient with a history of long-standing exertional breathlessness, presented with worsening symptoms rapidly evolving to orthopnea. A proper specific treatment was prescribed to the patient in the following days, including diuretics, angiotensin-converting-enzyme inhibitor and beta blockers. The patient died of progressive multiple organ failure.

**Conclusion:**

Echocardiography is technically limited due to the acoustic shadowing as a result of the calcification. Chest computed tomography is a more accurate diagnostic tool to examine the anatomic distribution and extent of endomyocardial calcification in this rare case.

## Background

In tropical countries of developing economy, endomyocardial fibrosis (EMF) is a rare disease, as well as a major cause of death. However, EMF is a major cause of restrictive cardiomyopathy with 10–12 million estimated cases worldwide [1]. EMF is characterized by impaired filling in one or both of the ventricles, resulted from the deposition of fibrous tissues on the surface of endocardium. For decades, the etiology of EMF has remained elusive, and there is no specific and/or effective treatment, both of which contribute to its poor prognosis. Multiple contributing factors have been proposed, including malnutrition, toxics, infectious agents, allergens and hyper-eosinophilia [2].

To date, cases of EMF have been mainly documented in South America and South Asia, where incidences were more frequently reported among impoverished young adults and children in developing countries [3, 4]. EMF is likely caused by the complex interaction of susceptible hosts exposed to environmental factors faced in case of severe poverty [5]. As a result, poor characterization and recognition at the early stage of this disease, as well as the lack of systematic research by modern scientific principles, have further hindered therapeutic discoveries for EMF.

## Case presentation

**Fig. 1:**
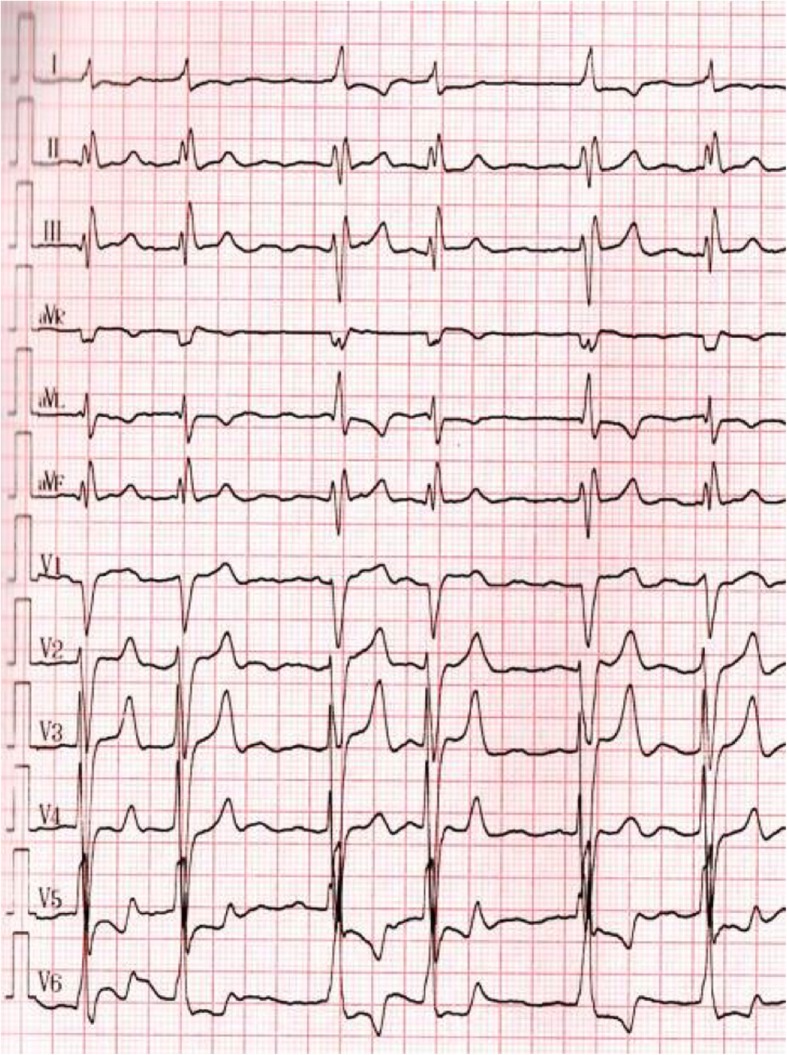
12 lead EKG shows atrial fibrillation with intraventricular block

**Fig. 2:**
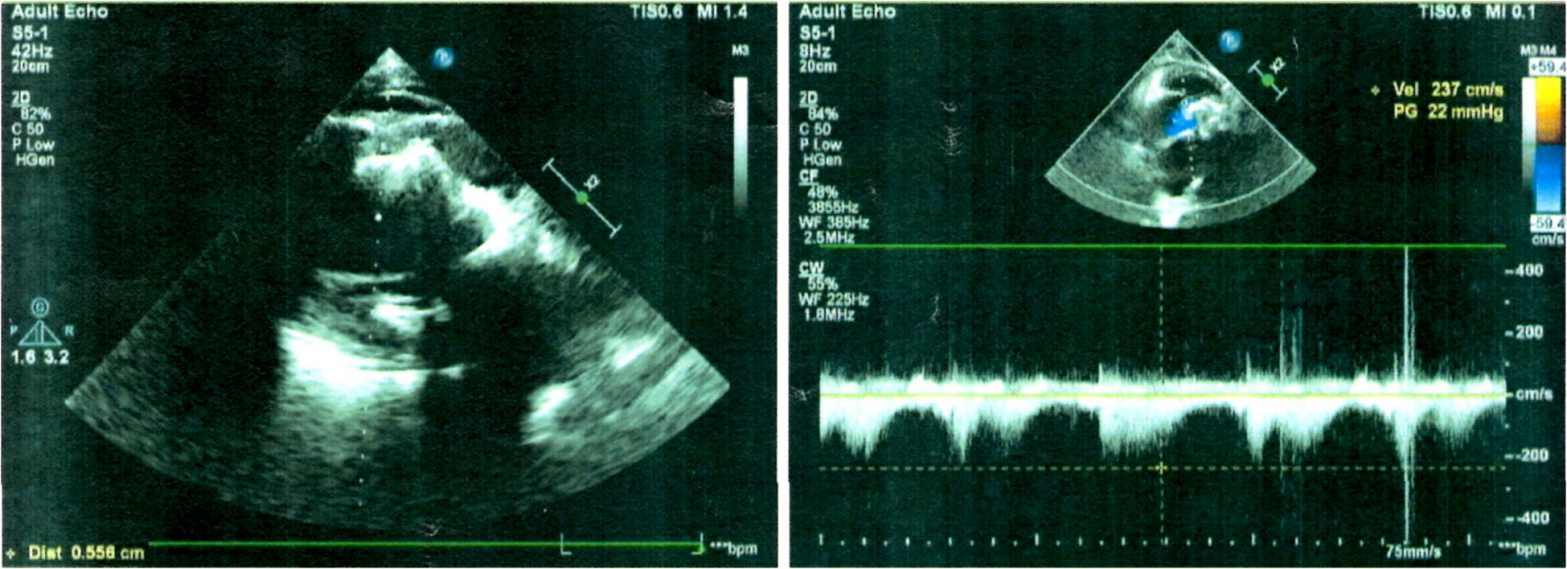
Echocardiography shows extensive endocardial calcification of the left ventricle

**Fig. 3:**
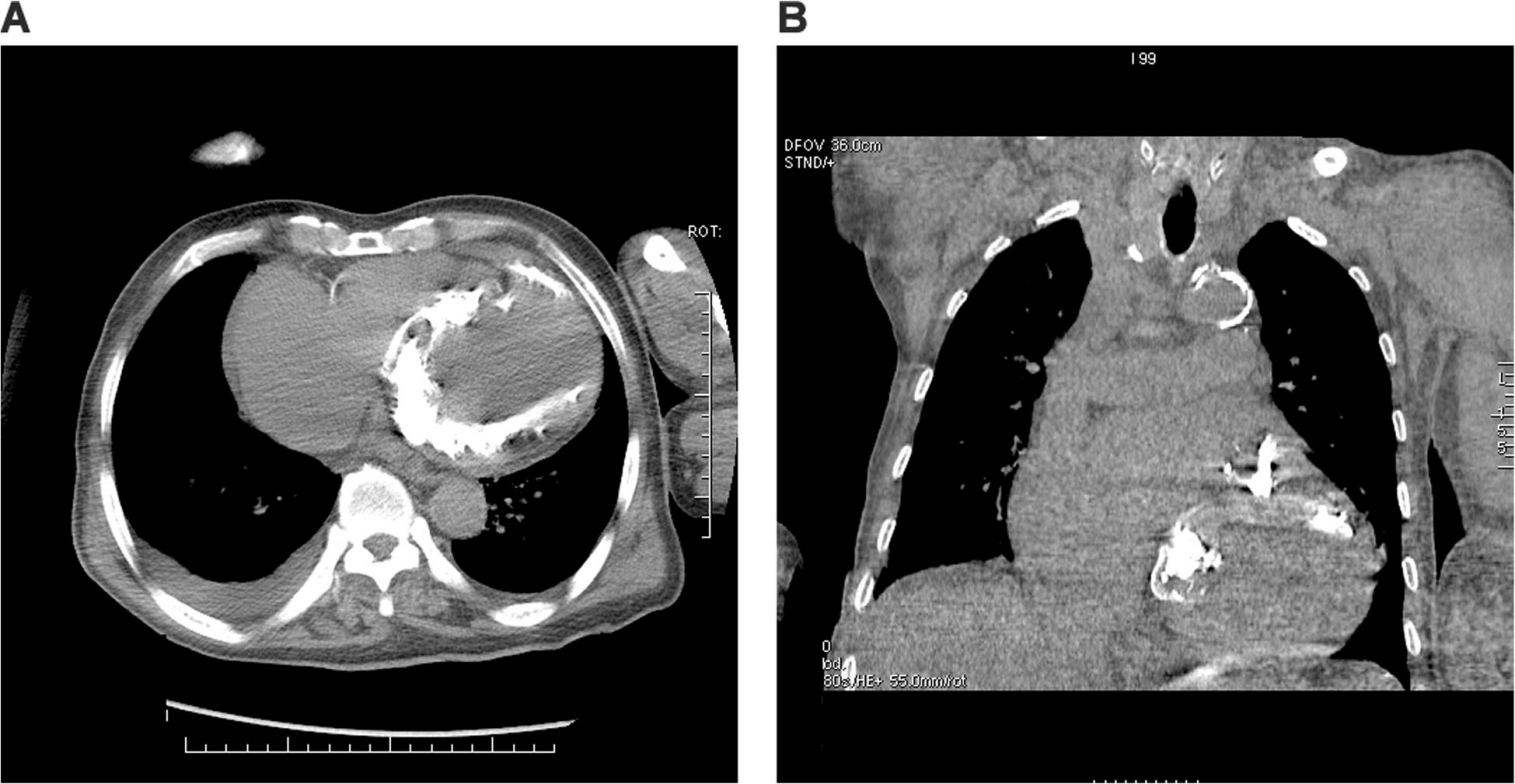
Computed tomography of chest, **a** Horizontal scan shows extensive calcification of left ventricular myocardium and interventricular septum. **b** Coronal scan with spots of patchy calcification on the left ventricle

An 81-year-old male Chinese patient with a history of long-standing exertional breathlessness, presented with worsening symptoms rapidly evolving to orthopnea, was admitted to our hospital in May 2016. At admission, patient was in New York Heart Association class III, with a blood pressure of 90/60 mmHg and a pulse of 120 beats/min. Physical examination showed mild bilateral ankle edema, muscle atrophy, jugular venous distension, hepatosplenomegaly, digital clubbing and mild pitting edema of lower extremities. At cardiac auscultation, a high-pitched pan-systolic mitral and tricuspid murmurs were clearly appreciated. Laboratory investigations revealed slightly elevated white blood cell count without hyper-eosinophilia, while electrocardiography showed atrial fibrillation and ST/T wave abnormalities (Fig. 1). On echocardiography, extensive endocardial calcification of the left ventricular apex and mitral valve apparatus were appreciable, with both atria dilated and left ventricular ejection fraction at 37% (Fig. 2). The mitral posterior leaflet was markedly hypoplastic and tethered to the ventricular wall, resulting in moderate-to-severe regurgitation. No significant pulmonary hypertension was detected. Computed tomography (CT) of the chest revealed a ‘shrunken’ left ventricle with heavy endomyocardial calcified patches at the level of the apex and lateral wall with both pericardial and pleural effusion (Fig. 3a and b).

Despite undergoing a proper specific treatment in the following days, including diuretics, angiotensin-converting-enzyme inhibitor and beta blockers, the patient died of heart failure and cardiogenic shock, followed by progressive multiple organ failure.

## Discussion and conclusion

Since first description in Uganda, high-incidence areas for EMF have been reported in Africa [6], India [7] and South America [8], and this disease seems to preferably afflict impoverished children and young adults in developing countries [3, 4].

In our present case, the patient presented with symptoms of cardiac failure, obliteration of the left ventricular apex, endomyocardial plaques, mitral regurgitation, enlarged left atrium and restrictive filling pattern, all of which are diagnostic criteria of EMF [3]. In addition, chest CT revealed severe calcification in the left ventricle. Biventricular involvement is the most typical presentation of EMF, followed by isolated right-sided heart disease. Among 55 cases studied by Vijayaraghavan et al. using echocardiography, 35 cases were found to have calcifications in ventricular walls, with right ventricular involvement in 26 cases and left ventricular involvement in 16 cases, respectively [9]. In sub-Saharan Africa, the disease is commonly right-sided or bilateral and rarely left-sided [6, 10]. On the other hand, as reported by Seth et al., the majority of EMF patients in India manifested at both ventricles [7]. In China, among the reported 87 cases of EMF in the southern part of the country, pathological and clinical features were similar to those in tropical areas with right ventricular involvement being the most frequent type [11]. However, massive calcification of isolated left ventricle, as observed in our current case by CT, is rare.

Our patient showed extensive myocardium calcification. There are two different pathophysiological mechanisms underlying myocardium calcification: dystrophic and metastatic calcification. Dystrophic calcification occurs in necrosis or degeneration of tissues, such as myocardial infarction, ventricular aneurysm, myocarditis and cardiac tumors. Calcified metastatic lesions occur in disorders of calcium metabolism. Due to the acoustic shadowing as a result of the calcified fibrosis, accuracy of echocardiography as a diagnostic tool is limited. On the other hand, chest CT is a more accurate diagnostic tool to examine the anatomic distribution and extent of endomyocardial calcification in this rare case. Canesin suggested that extensive myocardial calcification could be a clue for the diagnosis of EMF. The occurrence of endomyocardial calcification spots is usually a marker of burnt-out disease resulted from intermittent flare-ups of inflammation alternating with quiescent phases [9].

Furthermore, EMF can be mis-diagnosed as constrictive pericarditis or Ebstein’s anomaly: [12] 1) sub-valvular fibrosis in combination with apical obliteration could reduce the right ventricle cavity, leading to obvious downward displacement of the tricuspid valve, which could be exaggerated by a giant right atrium; 2) a patent foramen ovale stretched by tricuspid regurgitation could be mistakenly diagnosed as an atrial septal defect commonly seen in Ebstein’s anomaly. Echocardiography has become the mainstay of diagnosis for morphological analysis and non-invasive diagnostic assessment [3]. Magnetic resonance imaging (MRI), which is able to outline the degree of chamber distortion and the extent of thrombotic complications, also allows the differentiation of EMF from other conditions. Perfusion studies accurately map the hypo-perfused myocardial areas and avascular structures, whereas late-gadolinium-enhancement images correlate with fibrosis. Cardiac MRI would be an ideal tool for monitoring the response to treatment and for defining anatomic details before surgery. CT scans are more readily available but rarely used and may show the endocardial calcifications or the intracavitary thrombi.

Once diagnosed with EMF, the mean life expectancy of patients is 2 years, with direct causes of death being heart failure, arrhythmia and/or thromboembolic events. To date, there has been no specific and effective medical treatment, while surgery, including endo-cardectomy and valve repair/replacement, was reported to improve the prognosis [13]. Moraes et al. studied 83 cases of EMF patients with age ranging from 4 to 59 years and reported an operative mortality of approximately 18% [13]. After a following-up period ranging from 2 months to 17 years, only 45% of the surviving patients were in functional class I or II, while 5 patients received additional operation for recurrence of EMF.

### Learning points

It is worthy of noting that, most reported cases of EMF patients are much younger than our patient. The reason may be delayed seeking for health care, which is common in Chinese rural areas. Nevertheless, this case study of EMF patient presented interesting features including massive calcification of isolated left ventricle, normal absolute eosinophil count and its sporadic occurrence outside 15° of the equatorial belt (latitude 38°N), which prompts more cooperative research to reveal potential involving factors in various countries. This case highlighted the importance of adequate first-line medical attention in reducing mortality to EMF.

## Data Availability

Data could be obtained upon request to the corresponding author.

## Abbreviation

EMF: Endomyocardial fibrosis
